# Supplementary material for: Deep learning for high-throughput quantification of oligodendrocyte ensheathment at single-cell resolution
Source: Commun Biol. 2019 Mar 26;2:116. doi: 10.1038/s42003-019-0356-z (PMC6435748; doi:10.1038/s42003-019-0356-z)
Supplement: Supplementary file 1 — Supplemental Information [file 42003_2019_356_MOESM1_ESM.pdf]

SUPPLEMENTARY INFORMATION

Supplementary Tables

Supplementary Table 1: Average sheath lengths with standard deviation. Since the sheath length data was log normal (log lengths followed a Gaussian distribution), the average log length and standard deviation were used for statistical tests. The upper and lower quartile of the distribution are also reported in microns, along with the number of sheaths identified per condition.

Related figure	Condition	Mean log(length)	Standard deviation log(length)	Mean length (microns)	Number of Sheaths	25th Percentile (microns)	75th Percentile (microns)
3 A, B	UNet	1.58	0.29	48.84	461	21.63	63.94
3 A, B	H1	1.60	0.25	47.25	631	25.34	59.89
3 A, B	H2	1.60	0.25	47.58	497	26.69	57.68
3 A, B	H3	1.58	0.22	42.47	245	26.68	53.17
3 A, B	Heuristic	1.57	0.30	47.85	341	20.93	59.81
3 A, B	Ex1	1.68	0.23	54.25	477	33.75	65.83

Supplementary Table 2: Average sheath lengths with standard deviation. Since the sheath length data was log normal (log lengths followed a Gaussian distribution), the average log length and standard deviation were used for statistical tests. The upper and lower quartile of the distribution are also reported in microns, along with the number of sheaths identified per condition.

Related figure	Condition	Mean log(length)	Standard deviation log(length)	Mean length (microns)	Number of Sheaths	25th Percentile (microns)	75th Percentile (microns)
5 A, B	UNet, Laminin	1.597	0.31	51.43	18512	22.27	66.17
5 A, B	UNet, PDL	1.583	0.30	49.1	10490	21.71	63.23
5 A, B	Heuristic, Laminin	1.53	0.30	44.92	23264	19.16	54.87
5 A, B	Heuristic, PDL	1.52	0.30	42.33	13356	18.76	52.08
