# Supplementary material for: Deep learning for high-throughput quantification of oligodendrocyte ensheathment at single-cell resolution
Source: Commun Biol. 2019 Mar 26;2:116. doi: 10.1038/s42003-019-0356-z (PMC6435748; doi:10.1038/s42003-019-0356-z)
Supplement: Supplementary file 2 — Reporting Summary [file 42003_2019_356_MOESM2_ESM.pdf]

nature research

Corresponding author(s): Timothy E. Kennedy

Reporting Summary

Nature Research wishes to improve the reproducibility of the work that we publish. This form provides structure for consistency and transparency in reporting. For further information on Nature Research policies, see Authors & Referees and the Editorial Policy Checklist.

Statistical parameters

When statistical analyses are reported, confirm that the following items are present in the relevant location (e.g. figure legend, table legend, main text, or Methods section).

n/a	Confirmed	
☐	☒	The exact sample size (*n*) for each experimental group/condition, given as a discrete number and unit of measurement
☐	☒	An indication of whether measurements were taken from distinct samples or whether the same sample was measured repeatedly
☐	☒	The statistical test(s) used AND whether they are one- or two-sided<br>*Only common tests should be described solely by name; describe more complex techniques in the Methods section.*
☐	☒	A description of all covariates tested
☐	☒	A description of any assumptions or corrections, such as tests of normality and adjustment for multiple comparisons
☐	☒	A full description of the statistics including central tendency (e.g. means) or other basic estimates (e.g. regression coefficient) AND variation (e.g. standard deviation) or associated estimates of uncertainty (e.g. confidence intervals)
☐	☒	For null hypothesis testing, the test statistic (e.g. *F*, *t*, *r*) with confidence intervals, effect sizes, degrees of freedom and *P* value noted<br>*Give P values as exact values whenever suitable.*
☒	☐	For Bayesian analysis, information on the choice of priors and Markov chain Monte Carlo settings
☒	☐	For hierarchical and complex designs, identification of the appropriate level for tests and full reporting of outcomes
☒	☐	Estimates of effect sizes (e.g. Cohen's *d*, Pearson's *r*), indicating how they were calculated
☐	☒	Clearly defined error bars<br>*State explicitly what error bars represent (e.g. SD, SE, CI)*

Our web collection on statistics for biologists may be useful.

Software and code

Policy information about availability of computer code

Data collection	Automated acquisition of fluorescent images: Zen Black Systems 2.3 and Tiles and Positions software module (Zeiss)<br>Focus during acquisition of images: DefiniteFocus.2 module (Zeiss)<br>Generating training image masks: ImageJ 1.51n and MATLAB 2017
Data analysis	Convolutional neural network implementation: Tensorflow 1.6.0 and Python 3.1<br>Convolutional neural network training: Computer with NVIDIA Tesla P100-PCIE graphics card (Compute Canada)<br>Convolutional neural network testing: Computer with NVIDIA GTX 1070 graphics card, i7 CPU, 32 GB RAM (Dell)<br>Classic algorithmic approach implemented with MATLAB 2017<br>Statistical analysis: Graphpad prism 5<br>Russ Lenth's power calculator (Reference 16)

For manuscripts utilizing custom algorithms or software that are central to the research but not yet described in published literature, software must be made available to editors/reviewers upon request. We strongly encourage code deposition in a community repository (e.g. GitHub). See the Nature Research guidelines for submitting code & software for further information.

Data

Policy information about availability of data

All manuscripts must include a data availability statement. This statement should provide the following information, where applicable:

- Accession codes, unique identifiers, or web links for publicly available datasets
- A list of figures that have associated raw data
- A description of any restrictions on data availability

The datasets generated during and/or analysed during the current study are available from the corresponding author on request.

Field-specific reporting

Please select the best fit for your research. If you are not sure, read the appropriate sections before making your selection.

☒ Life sciences ☐ Behavioural & social sciences ☐ Ecological, evolutionary & environmental sciences

For a reference copy of the document with all sections, see nature.com/authors/policies/ReportingSummary-flat.pdf

Life sciences study design

All studies must disclose on these points even when the disclosure is negative.

Sample size	Sample size was based on the experience of the authors and in similar published research, to ensure adequate statistical power. In addition, the sample sizes for each experiment have been detailed and confirmed statistically by appropriate power tests.
Data exclusions	No data was excluded from the analyses.
Replication	Performance of the algorithms was verified by replication with multiple human researchers and different machine approaches. We also successfully replicated the findings of Bechler et al. (2015) for PDL and Laminin coating effects on ensheathment properties.
Randomization	Randomization was not relevant to the study.
Blinding	Investigators were blinded to coating type during ensheathment analysis and researcher bias was further removed by retaining the same analytic parameters across each experiment with the machine programs.

Reporting for specific materials, systems and methods

Materials & experimental systems

n/a	Involved in the study
☒	☐ Unique biological materials
☐	☒ Antibodies
☒	☐ Eukaryotic cell lines
☒	☐ Palaeontology
☐	☒ Animals and other organisms
☒	☐ Human research participants

Methods

n/a	Involved in the study
☒	☐ ChIP-seq
☒	☐ Flow cytometry
☒	☐ MRI-based neuroimaging

Antibodies

Antibodies used	Antibody protocols and catalogue numbers are provided in the methods sections.<br><br>Chicken anti-Myelin Basic Protein (1:1000, Aves Labs, #MBP)<br>Alexa 546 Goat Anti-Chicken (1:1000, ThermoFisher, #A-11040)
Validation	The MBP antibody undergoes IHC quality control testing (http://www.aveslab.com/products/glial-and-schwann-cell-markers/mbp-myelin-basic-protein-chicken-polyclonal-anti-peptide-antibody/) and was used in a previous publication on knock-out tissue cultures which validated its specificity (https://doi.org/10.1371/journal.pone.0041237).

Animals and other organisms

Policy information about studies involving animals; ARRIVE guidelines recommended for reporting animal research

Laboratory animals	Female Sprague-Dawley rats with 2-day old litters were ordered from Charles River. Cells isolated from each litter were pooled. Animal experiments were performed under protocol 2001-4330, in accordance with the Canadian Council on Animal Care guidelines for the use of animals in research and approved by the Montreal Neurological Institute Animal Care Committee and the McGill Animal Compliance Office.
Wild animals	This study did not involve wild animals.
Field-collected samples	This study did not involve samples collected from the field.
